# Mainstreaming neglected tropical disease interventions into primary health care: accelerating elimination and sustaining gains

**DOI:** 10.1016/j.lanprc.2026.100177

**Published:** 2026-06

**Authors:** Kebede Deribe, Esmael Habtamu

**Affiliations:** Children’s Investment Fund Foundation, Addis Ababa, Ethiopia; Centre for Equitable Global Health Research, Brighton and Sussex Medical School, Brighton BN1 9PX, UK; International Centre for Eye Health, Clinical Research Department, London School of Hygiene & Tropical Medicine, London, UK; Eyu-Ethiopia, Eye Health Research, Training and Service Centre, Bahir Dar, Ethiopia; College of Medicine and Health Science, Bahir Dar University, Bahir Dar, Ethiopia

Neglected tropical diseases (NTDs) are a group of conditions that disproportionately affect people living in deprived areas with limited access to water, sanitation, and health services.^1^ These diseases impose substantial health, social, and economic burdens, affecting around 1 billion people globally.^2^ The WHO NTD Roadmap for 2021–30 aims to reduce the number of people requiring treatment by 90% and disability-adjusted life-years by 75%, eliminate at least one NTD in 100 countries, and eradicate two diseases (dracunculiasis and yaws)^1^ through five core strategies: preventive chemotherapy; intensified disease management; vector control; veterinary public health; and water, sanitation, and hygiene (WASH).

Considerable progress has been made during the past decade. The number of people requiring interventions declined from 2·19 billion in 2010 to 1·495 billion in 2023 (a 32% reduction) and, as of April, 2026, 63 countries have eliminated at least one NTD.^3^ Much of this progress has relied on large-scale, time-bound delivery strategies, commonly implemented as campaigns, supported by external partners, and delivered through frontline health workers.^3^ As countries approach elimination, a key question remains: how can these approaches be aligned with primary health care (PHC) systems to both achieve elimination and sustain gains?

Mainstreaming NTD interventions does not mean replacing campaigns but embedding them within core health-system functions, including governance, financing, service delivery, workforce, information systems, and supply chains (table).^4^ For several NTDs, campaign-based delivery is epidemiologically indispensable.^5^ Preventive chemotherapy, for example, depends on reaching large populations within short timeframes through campaigns, something that routine PHC services alone cannot achieve. Campaign-based delivery should therefore be viewed as an extension of the health system, expanding the reach and surge capacity of PHC to serve large and remote populations efficiently.^6^ However, as disease prevalence declines, programme priorities shift towards surveillance, case detection, management of chronic conditions, and prevention of resurgence.^1^ These functions require services that are continuously available and embedded within PHC and community platforms. In this context, interventions are mainstreamed when they are delivered through and reinforce core health-system functions rather than relying on parallel or vertical arrangements. Advancing this approach depends on three interrelated shifts.

First, stronger national ownership is needed, with governments leading the planning, coordination, financing, and delivery of NTD programmes, including campaigns. Evidence suggests that mainstreaming is often more advanced at the district level than at national and regional levels, where weak governance, fragmented coordination, and limited managerial capacity remain major barriers.^7,8^ National ownership should therefore extend beyond policy endorsement to include operational leadership, domestic resource mobilisation, and accountability for programme performance. However, greater national ownership does not imply reduced collaboration with external partners but a rebalancing of roles in which international and local support are aligned behind nationally defined priorities.

Second, mainstreaming requires sustained investment in health-system capacity, ensuring that NTD financing strengthens systems rather than supporting isolated programme outputs. This shift calls for government-led mechanisms that deliberately channel NTD investments into workforce development, supply-chain strengthening, digital health information systems, managerial capacity, and community platforms. Campaign financing should therefore be designed to not only deliver short-term coverage targets but also create stronger system capacity for routine service delivery. Without sustained domestic financing and deliberate investment in system strengthening, mainstreaming will remain rhetorical rather than operational.

Third, mainstreaming should be differentiated across intervention types, recognising that one model of integration will not fit all NTD activities. Preventive chemotherapy campaigns can increasingly be implemented as key and planned extensions of the PHC system, with frontline health workers and local health structures leading the implementation. Intensified disease management can be more fully embedded within routine PHC and community-based services through training of frontline health workers, digitalised clinical pathways, and community sensitisation. Vector control and WASH interventions should be coordinated through intersectoral platforms linked to broader development systems. Surveillance should be integrated into national information systems and community reporting structures, with increasing sensitivity as elimination approaches. Ethiopia’s electronic community health information system offers a promising platform to integrate NTD screening referral, follow-up, and reporting within routine PHC workflows, strengthening continuity of care, data visibility, and programme responsiveness.^9,10^ Mainstreaming is therefore best understood not as a single model but as a continuum of differentiated approaches shaped by the nature of the health challenge, complexity of the intervention, health-system capacity, and broader country contexts, as well as the extent to which interventions are embedded within core health-system functions.^4^

As countries move closer to eliminating NTDs, the key challenge shifts from scaling interventions to sustaining progress. Campaigns are not the opposite of mainstreaming; poorly aligned ones are. Campaign-based delivery continues to be important for reducing transmission and addressing remaining gaps,^6^ but its long-term value depends on how well it is incorporated into the structures and processes of the health system as a key extension of PHC. When properly aligned with national priorities and system strengthening, campaign-based approaches could become complementary components of a coherent and mainstreamed strategy that would not only accelerate elimination but also build stronger and more resilient health systems.

## Figures and Tables

**Table:** Characteristics of non-mainstreamed versus mainstreamed delivery of neglected tropical disease interventions across health-system domains

	Non-mainstreamed model	Mainstreamed model
Governance	Interventions coordinated by external partners or non-governmental organisations with parallel structures, minimal national oversight	Interventions planned, led, and coordinated by Ministries of Health within national and subnational health plans, with clear accountability and governance mechanisms
Financing	Fully donor-funded, off-budget, disease-specific funding streams	Progressive domestic co-financing, donor funding pooled or aligned with national financing systems and priorities
Service delivery	Delivered through standalone campaign teams with weak linkage to routine primary health care services	Delivered through existing primary health care and community platforms, aligned with routine outreach and service-delivery mechanisms
Health workforce	Temporary or parallel workforce with campaign-specific roles and little continuity	Routine health workforce (including community health workers) delivering interventions, training-based strengthening of competencies beyond campaign-based approaches
Medicines and technologies	Separate procurement and distribution systems for campaign and other intervention commodities, partial integration with routine logistics	Commodities procured, distributed, and managed through national supply chains, including reverse logistics and stock management
Health information systems	Parallel data collection and reporting systems used primarily for donor reporting, little use in national decision making	Data captured within national information systems and used for routine planning, surveillance, and response
Community systems	One-off mobilisation for campaign delivery with reduced continuity or engagement beyond the campaign period	Engagement through existing community structures, supporting sustained participation in case detection, referral, and follow-up
